# Accumulation of Deleterious Mutations Near Sexually Antagonistic Genes

**DOI:** 10.1534/g3.116.031161

**Published:** 2016-05-24

**Authors:** Tim Connallon, Crispin Y. Jordan

**Affiliations:** * School of Biological Sciences, Monash University, Clayton, Victoria 3800, Australia; † Ashworth Laboratories, Institute of Evolutionary Biology, The University of Edinburgh, EH9 3JT, UK

**Keywords:** intralocus sexual conflict, genetic load, haploid selection, recombination, linkage disequilibrium

## Abstract

Mutation generates a steady supply of genetic variation that, while occasionally useful for adaptation, is more often deleterious for fitness. Recent research has emphasized that the fitness effects of mutations often differ between the sexes, leading to important evolutionary consequences for the maintenance of genetic variation and long-term population viability. Some forms of sex-specific selection—*i.e.*, stronger purifying selection in males than females—can help purge a population’s load of female-harming mutations and promote population growth. Other scenarios—*e.g.*, sexually antagonistic selection, in which mutations that harm females are beneficial for males—inflate genetic loads and potentially dampen population viability. Evolutionary processes of sexual antagonism and purifying selection are likely to impact the evolutionary dynamics of different loci within a genome, yet theory has mostly ignored the potential for interactions between such loci to jointly shape the evolutionary genetic basis of female and male fitness variation. Here, we show that sexually antagonistic selection at a locus tends to elevate the frequencies of deleterious alleles at tightly linked loci that evolve under purifying selection. Moreover, haplotypes that segregate for different sexually antagonistic alleles accumulate different types of deleterious mutations. Haplotypes that carry female-benefit sexually antagonistic alleles preferentially accumulate mutations that are primarily male harming, whereas male-benefit haplotypes accumulate mutations that are primarily female harming. The theory predicts that sexually antagonistic selection should shape the genomic organization of genetic variation that differentially impacts female and male fitness, and contribute to sexual dimorphism in the genetic basis of fitness variation.

Mutation generates a steady influx of new genetic variation. Nonneutral mutations primarily reduce fitness, and are consequently kept rare within a population by purifying natural selection (Lynch *et al.* 1999; Eyre-Walker and Keightley 2007). Nevertheless, the cumulative fitness cost – referred to as the “genetic load” – of individually rare deleterious alleles is likely to be substantial (Crow and Kimura 1970; Simmons and Crow 1977; Halligan and Keightley 2009; Agrawal and Whitlock 2012), and this variation is expected to contribute to inbreeding depression, reduced capacity for population growth, and heightened extinction risk (Haldane 1937; Charlesworth and Charlesworth 1987; Lynch and Gabriel 1990).

Research during the past decade has increasingly emphasized the role of sex differences in selection on population genomic diversity, including the maintenance of genetic variation that contributes to the distinct genetic loads of females and males (Whitlock and Agrawal 2009; Connallon *et al.* 2010; Mallet and Chippindale 2011; Ebel and Phillips 2016). The presence of separate sexes (or sex functions) is thought to influence the genetic load of each sex in at least three ways: (1) through sex differences in the fitness costs of deleterious mutations; (2) through sexually antagonistic genetic variation; and (3) through sex differences in the mutation rate. We describe each of these effects below.

Deleterious mutations often impact the sex roles unequally, with mutations that strongly affect the fitness of one sex, modestly affecting fitness of the other (*e.g.*, Whitlock and Agrawal 2009; Connallon and Clark 2011). Under some circumstances, sex differences in selection against deleterious alleles can reduce the female genetic load, at the expense of males, and fortuitously improve population growth (Manning 1984; Agrawal 2001; Siller 2001). This scenario arises when deleterious alleles are, on average, more harmful to male fitness than to female fitness – a pattern that has been repeatedly documented in lab populations of *Drosophila* (Sharp and Agrawal 2008, 2013; Hollis *et al.* 2009; Mallet *et al.* 2011), though less consistently in other animal systems (*e.g.*, Almbro and Simmons 2014; Power and Holman 2015). Sex-limited deleterious mutations, which are harmful for one sex and neutral for the other, represent an extreme class of mutations with unequal deleterious effects between the sexes. Such mutations readily accumulate within populations due to being sheltered from purifying selection within one of the two sexes (Van Dyken and Wade 2010; Connallon *et al.* 2010); the accumulation of both female- and male-limited mutations within a population is expected to elevate the genetic loads of both sexes (see Day and Bonduriansky 2004; Connallon *et al.* 2010).

Sexually antagonistic mutations, which individually improve fitness within one sex while reducing it in the other (see Rice 1992), represent an additional source of genetic variation for fitness in animal and plant populations (Chippindale *et al.* 2001; Delph *et al.* 2004; Foerster *et al.* 2007; Abbott 2011). Sexually antagonistic alleles are expected to segregate at relatively high population frequencies compared to unconditionally deleterious mutations (Connallon and Clark 2012). Some sexually antagonistic alleles may be stably maintained at intermediate frequencies by balancing selection (*e.g.*, Kidwell *et al.* 1977; Jordan and Connallon 2014; Tazzyman and Abbott 2015), which occurs most readily when the strength of antagonistic selection is strong and roughly equal between the sexes (Kidwell *et al.* 1977; Patten and Haig 2009; Fry 2010). In such cases, sexually antagonistic alleles will similarly inflate the genetic loads of both sexes (Whitlock and Agrawal 2009; Connallon *et al.* 2010).

Finally, sex differences in selection appear to indirectly favor the evolution of male-biased mutation rates, which potentially elevate the total mutation rate in species with separate sexes (Ellegren 2007; Hedrick 2007; Whitlock and Agrawal 2009). Male-biased mutation rates are common among vertebrates (Ellegren 2007; Hedrick 2007), and are occasionally reported within invertebrates (Bachtrog 2008). Many species exhibit a twofold or higher mutation rate in males than females, and some taxa exceed ratios of 8- to 10-fold (Hedrick 2007). While male-biased mutation can potentially elevate the total deleterious mutation rate of a species, and thereby increase the average load of deleterious mutations per genome, this theoretical prediction currently lacks direct empirical support, and remains open to investigation (for discussion, see Whitlock and Agrawal 2009).

Here, we demonstrate that sexually antagonistic genetic variation, sexually asymmetric selection against deleterious alleles, and male-biased mutation rates interact to shape the burden of deleterious mutations that are carried by individuals of a population. Inspired by recent theoretical work on multilocus balancing selection on sexually antagonistic alleles (Patten *et al.* 2010, 2013; Úbeda *et al.* 2011; Haig *et al.* 2014; Patten 2014), we demonstrate that sexually antagonistic polymorphism invariably promotes the accumulation of deleterious alleles in genes that are physically linked to sexually antagonistic loci. The interaction between sexual antagonism and linkage arises from the unequal transmission patterns of sexually antagonistic variation, where male-beneficial alleles are preferentially transmitted from fathers to offspring, and female-beneficial alleles are preferentially maternally inherited (Day and Bonduriansky 2004; Patten 2014). This transmission bias mediates the strength of selection against deleterious mutations that cosegregate with each sexually antagonistic allele. We show that female-limited deleterious alleles readily accumulate in tight linkage with male-benefit sexually antagonistic alleles. Likewise, male-limited deleterious mutations accumulate near antagonistic alleles with female-beneficial effects. We also show that sexually antagonistic polymorphism distorts the mutation rates of linked genes, with elevated mutation rates upon haplotypes that carry male-benefit sexually antagonistic alleles. The theory predicts an elevated deleterious mutation load at loci that are linked to sexually antagonistic genes, with haplotypes that segregate for sexually antagonistic alleles each enriched in deleterious mutations that are particularly damaging for one sex and relatively benign for the other.

## The Model

We track the evolution of a pair of biallelic loci with arbitrary degree of physical linkage. Locus *A* is evolving under sexually antagonistic selection, with a male-beneficial allele (*A*_m_) and female-beneficial allele (*A*_f_). We assume that *A*_f_ and *A*_m_ are maintained by balancing selection, and ignore effects of mutation at the locus. Locus *B* segregates for two alleles: *B*_1_ is a wild-type allele with relative fitness of one, and *B*_2_ is a deleterious mutation that is maintained at a balance between recurrent mutation and purifying selection to remove it from the population. For simplicity, we assume that the per gamete mutation rate from *B*_1_ to *B*_2_ is *u*_f_ in females and *u*_m_ in males, with no back-mutation. We also assume that the pair of loci (*A* and *B*) affect fitness multiplicatively. The loci are separated by a recombinational distance of *r* (0 < *r* < 1/2), which reflects the probability of a recombination event between the loci, per meiosis. Generations are discrete, and follow the order of: (i) birth, (ii) viability selection, (iii) gametogenesis including mutation, and (iv) random mating and reproduction among the adults.

We consider two inheritance models – haploidy and diploidy – that arise often within dioecious animal and plant species. For the haploid model – applicable, for example, in primitive plant species (see Jesson and Garnock-Jones 2012) and algae – meiosis occurs shortly after fertilization, and individuals spend most of their life cycle within the haploid stage. Consequently, selection occurs among haploid individuals, and genetic dominance does not factor into the evolutionary dynamics at either locus. In contrast, for the diploid model, which applies in many dioecious animals and higher plants, meiosis co-occurs with the production of gametes, selection acts among diploid individuals, and dominance becomes critically important in the dynamics of both sexually antagonistic and deleterious alleles (but see Immler *et al.* 2012; Otto *et al.* 2015).

For each model, we follow the evolution of four haplotypes that segregate (within zygotes) at frequencies: *x*_1_ = [*A*_m_*B*_1_], *x*_2_ = [*A*_m_*B*_2_], *x*_3_ = [*A*_f_*B*_1_], and *x*_4_ = [*A*_f_*B*_2_]. Recursion equations follow from standard population genetics models of sex-specific selection (*e.g.*, Kidwell *et al.* 1977; Connallon and Clark 2010), and are presented in full within the *Appendix*. The sex-specific fitness values for each of the resulting four haploid genotypes and nine diploid genotypes are presented in Table 1. Since we are primarily concerned with the overall frequency of deleterious mutations within the population, and the relative frequency of mutations on *A*_f_-bearing and *A*_m_-bearing haplotypes, our analysis focuses on each of the two mutant-bearing haplotypes: the *A*_m_*B*_2_ haplotype at frequency *x*_2_, and the *A*_f_*B*_2_ haplotype at frequency *x*_4_. The total frequency of the deleterious allele is simply their sum: *x*_2_ + *x*_4_.

**Table 1:** Sex-specific fitness for each genotype (*w*_f_ and *w*_m_ for females and males, respectively)

	*A*_m_, *A*_m_*A*_m_	*A*_m_*A*_f_	*A*_f_, *A*_f_*A*_f_
*B*_1_, *B*_1_*B*_1_	*w*_f_ = 1 – *s*_f_	*w*_f_ = 1 – *k*_f_*s*_f_	*w*_f_ = 1
	*w*_m_ = 1	*w*_m_ = 1 – *k*_m_*s*_m_	*w*_m_ = 1 – *s*_m_
*B*_1_*B*_2_	*w*_f_ = (1 – *s*_f_)(1 – *ht*_f_)	*w*_f_ = (1 – *k*_f_*s*_f_)(1 – *ht*_f_)	*w*_f_ = 1 – *ht*_f_
	*w*_m_ = 1 – *ht*_m_	*w*_m_ = (1 – *k*_m_*s*_m_)(1 – *ht*_m_)	*w*_m_ = (1 – *s*_m_)(1 – *ht*_m_)
*B*_2_, *B*_2_*B*_2_	*w*_f_ = (1 – *s*_f_)(1 – *t*_f_)	*w*_f_ = (1 – *k*_f_*s*_f_)(1 – *t*_f_)	*w*_f_ = 1 – *t*_f_
	*w*_m_ = 1 – *t*_m_	*w*_m_ = (1 – *k*_m_*s*_m_)(1 – *t*_m_)	*w*_m_ = (1 – *s*_m_)(1 – *t*_m_)

For the sexually antagonistic locus (locus *A*), there are four parameters: sex-specific selection coefficients, *s*_f_ and *s*_m_, against the deleterious allele for each sex, and dominance coefficients, *k*_f_ and *k*_m_ (0 < *s*_m_, *s*_f_ < 1; 0 < *k*_m_, *k*_f_ < 1). We use three parameters for the *B* locus: sex-specific selection coefficients, *t*_m_ and *t*_f_, for the cost of the deleterious allele (*B*_2_), and a single dominance coefficient (*h*) with respect to *B*_2_, which we assume does not differ between the sexes (0 < *t*_m_, *t*_f_, *h* < 1).

We adopt two approaches in analyzing the polymorphic equilibrium for each model of inheritance. We first develop a set of relatively simple analytical approximations for the case of tight linkage (*r* → 0; see the *Appendix*), and assuming weak mutation relative to the strength of purifying selection (*i.e.*, *u*_m_ + *u*_f_ << *t*_m_ + *t*_f_). Second, we simulate exact equilibrium conditions under arbitrary mutation, selection, and recombination between loci. The case of tight linkage serves as a useful benchmark that provides an upper limit for the effect of sexually antagonistic polymorphism on the accumulation of deleterious mutations. As we later show, the effect of sexually antagonistic polymorphism on deleterious alleles predictably diminishes as the recombination rate increases. Ultimately, standard mutation-selection balance approximations (*e.g.*, Haldane 1937; Crow and Kimura 1970) apply as linkage becomes loose (*e.g.*, *r* → 1/2).

### Data availability

The authors state that all data necessary for confirming the conclusions presented in the article are represented fully within the article.

## Results

We subdivide our results into three main sections. We first develop a general model that characterizes the qualitative effect of sexually antagonistic polymorphism on the evolution of a tightly linked locus evolving under purifying selection. Second, we develop haploid- and diploid-specific models of sexually antagonistic polymorphism to quantify deleterious mutation accumulation near sexually antagonistic genes; this once again assumes tight linkage between loci. Third, we present simulation results that evaluate the interaction between sexually antagonistic loci and partially linked deleterious alleles (*e.g.*, under modest recombination rates between loci: 0 < *r* < 1/2).

### Mutation accumulation under tight linkage to a sexually antagonistic locus

Under tight linkage between locus *A* and locus *B*, and weak mutation to the deleterious allele (*r*, *u*_m_, *u*_f_ → 0), the equilibrium frequencies of *B*_2_-bearing haplotypes are:

$${\hat{x}}_{2}\approx C\hat{q}\frac{2\bar{u}+\frac{\delta }{2\hat{q}}\left(u_{\text{m}}-u_{\text{f}}\right)}{2\bar{t}-\frac{\delta }{2\hat{q}}\left(t_{\text{f}}-t_{\text{m}}\right)},$$ (1a)

and

$${\hat{x}}_{4}\approx C\left(1-\hat{q}\right)\frac{2\bar{u}-\frac{\delta }{2\left(1-\hat{q}\right)}\left(u_{\text{m}}-u_{\text{f}}\right)}{2\bar{t}+\frac{\delta }{2\left(1-\hat{q}\right)}\left(t_{\text{f}}-t_{\text{m}}\right)},$$ (1b)

where $\bar{t}=\left(t_{\text{m}}+t_{\text{f}}\right)/2$ is the sex-averaged selection coefficient against the *B*_2_ allele, $\bar{u}=\left(u_{\text{m}}+u_{\text{f}}\right)/2$ is the sex-averaged mutation rate to the *B*_2_ allele, $\hat{q}$ = [*A*_m_] is the equilibrium frequency of the male-benefit sexually antagonistic allele, *δ* = [(*A*_m_ | adult males) – (*A*_m_ | adult females)] is the frequency difference of the *A_m_* allele between breeding males and females, and *C* is a constant that accounts for ploidy level (*C* = 1 for haploids; *C* = 1/*h* for diploids). Note that all five terms must take positive values (*C*, *δ*, *u*, *t*, *q* > 0).

Sexually antagonistic polymorphism influences the cosegregation patterns of linked deleterious alleles. Equations (1a, b) can be used to quantify an individual’s probability of inheriting a deleterious mutation, given their allelic status at a sexually antagonistic locus. The “relative risk” of inheriting a deleterious mutation, given inheritance of a male- *vs.* a female-benefit allele at the sexually antagonistic locus, is:

$$RR=\frac{\mathrm{Pr}\left(B_{2}|A_{\text{m}}\right)}{\mathrm{Pr}\left(B_{2}|A_{\text{f}}\right)}=\frac{{\hat{x}}_{2}/\hat{q}}{{\hat{x}}_{4}/\left(1-q\right)}\approx 1+\frac{\delta }{4\hat{q}\left(1-\hat{q}\right)}\left[\frac{\left(u_{\text{m}}-u_{\text{f}}\right)}{\bar{u}}+\frac{\left(t_{\text{f}}-t_{\text{m}}\right)}{\bar{t}}\right]+O(\delta^{2}),$$ (2)

which follows from the similar concept of relative risk from epidemiology (see Jewell 2004, chapter 4). *RR* is scaled so that *RR* > 1 corresponds to an enrichment of the deleterious allele on haplotypes that carry the male-beneficial allele (*A*_m_); *RR* < 1 corresponds to enrichment of deleterious alleles on the female-beneficial haplotype (*A*_f_). The final approximation, to first order in *δ*, shows that deleterious alleles tend to preferentially segregate on *A_m_*-bearing haplotypes when their fitness effects are more severe in females than males (*t*_f_ > *t*_m_), and when mutation rates are higher in males than females (*u*_m_ > *u*_f_). Taking both factors into account, deleterious alleles in which *t*_f_/*t*_m_ > *u*_f_/*u*_m_ will be enriched on *A_m_*-bearing haplotypes, and alleles in which *t*_m_/*t*_f_ > *u*_m_/*u*_f_ will be enriched on *A*_f_-bearing haplotypes. When mutation rates are male-biased, the first of the two conditions is the most permissible, and under this scenario, deleterious mutations should preferentially accumulate on haplotypes that carry male-benefit sexually antagonistic alleles.

Sexually antagonistic polymorphism also alters the total frequency of deleterious alleles in the population. In the absence of sexually antagonistic selection, the equilibrium frequency of the deleterious allele is ${\hat{q}}_{B_{2}}={\hat{x}}_{2}+{\hat{x}}_{4}\approx C\left(\bar{u}/\bar{t}\right)$, which corresponds to the standard result under mutation-selection balance. Linkage to a sexually antagonistic locus modifies the mutation-selection equilibrium to:

$$\begin{array}{c} {\hat{x}}_{2}+{\hat{x}}_{4}\approx C\hat{q}\frac{2\bar{u}+\frac{\delta }{2\hat{q}}\left(u_{\text{m}}-u_{\text{f}}\right)}{2\bar{t}-\frac{\delta }{2\hat{q}}\left(t_{\text{f}}-t_{\text{m}}\right)}+C\left(1-q\right)\frac{2\bar{u}-\frac{\delta }{2\left(1-\hat{q}\right)}\left(u_{\text{m}}-u_{\text{f}}\right)}{2\bar{t}+\frac{\delta }{2\left(1-\hat{q}\right)}\left(t_{\text{f}}-t_{\text{m}}\right)}\\ =C\left[\frac{\bar{u}}{t}+\delta^{2}\frac{\left(t_{\text{f}}-t_{\text{m}}\right)\left(t_{\text{f}}u_{\text{m}}-t_{\text{m}}u_{\text{f}}\right)}{16\hat{q}\left(1-\hat{q}\right){\bar{t}}^{3}}\right]+O(\delta^{3}). \end{array}$$ (3)

Equation (3) provides insight into the effects of sexually antagonistic polymorphism on the accumulation of deleterious alleles. Linkage to a sexually antagonistic locus has no effect on the overall frequency of a deleterious allele unless the strength of purifying selection differs between the sexes (*i.e.*, *t*_m_ ≠ *t*_f_). When purifying selection is asymmetric between the sexes, the overall frequency of the deleterious allele can potentially increase or decrease relative to standard theory of mutation-selection balance. The deleterious allele decreases in frequency when (1 – *t*_m_/*t*_f_)(*u*_m_/*u*_f_ – *t*_m_/*t*_f_) < 0, and it increases when (1 – *t*_m_/*t*_f_)(*u*_m_/*u*_f_ – *t*_m_/*t*_f_) > 0. Although sexual antagonism can, in principle, increase or decrease the total frequency of linked deleterious alleles, the former is a much more likely outcome than the latter. A decrease in frequency is only possible for mutations that carry a higher fitness cost in the sex with the higher mutation rate, but even in such cases, conditions remain restrictive for a net decrease of deleterious allele frequency (a net decrease requires that one of the following conditions is true: either *u*_m_/*u*_f_ > *t*_m_/*t*_f_ > 1 or *u*_f_/*u*_m_ > *t*_f_/*t*_m_ > 1). These conditions will be difficult to meet when mutation rates are similar between sexes, or when deleterious mutations have strongly asymmetric effects on male and female fitness. For example, mutations with sex-limited fitness effects always increase in frequency when linked to a sexually antagonistic locus.

### Sexual antagonism and mutation accumulation in haploid and diploid species

Sexually antagonistic allele frequency differences between adult males and females favor the accumulation of deleterious alleles within a population, as demonstrated above [*i.e.*, equation (3)]. Such allele frequency differences between sexes also give rise to biases in the mutation load linked to male- *vs.* female-beneficial sexually antagonistic alleles [*i.e.*, equation (2)]. The magnitude of these effects scales positively with the degree of the sexually antagonistic allele frequency differentiation between adult males and females, represented by *δ* = [(*A*_m_ | adult males) – (*A*_m_ | adult females)]. To quantify effects of sexually antagonistic polymorphism on mutation accumulation, we develop expressions for *δ* under haploid and diploid models of sexually antagonistic selection. We then use these expressions to identify scenarios of antagonistic polymorphism that are most favorable for deleterious mutation accumulation. We start with expressions for *δ* for the haploid case, and then consider diploids. We then use each result to quantify effects of sexually antagonistic alleles on linked deleterious mutations.

#### Sexually antagonistic variation in haploids:

Under a haploid model of balancing selection at a sexually antagonistic locus, the equilibrium frequency of the male-beneficial allele (in zygotes) is:

$${\hat{q}}_{\text{hap}}=\frac{s_{\text{m}}-s_{\text{f}}+s_{\text{m}}s_{\text{f}}}{2s_{\text{m}}s_{\text{f}}}.$$ (4a)

**Figure 1:**
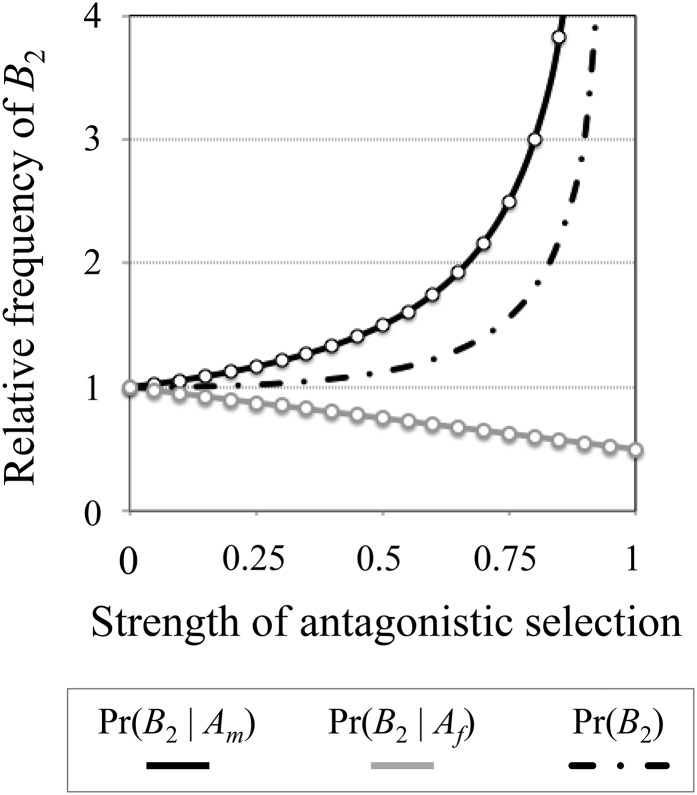
Deleterious mutation accumulation near a sexually antagonistic locus under haploid selection. Results show the relative frequency of a deleterious mutation (*B*_2_) with a female-limited fitness effect. The deleterious allele is linked to a sexually antagonistic locus, and selection occurs in the haploid state. The *x*-axis shows the strength of sexually antagonistic selection, which in this case is assumed to be equal between the sexes (0 < *s*_f_ = *s*_m_ < 1). Results on the *y*-axis are scaled relative to its equilibrium frequency of the deleterious allele in the absence of linkage: *p*_null_ = (*u*_f_ + *u*_m_)/(*t*_f_ +*t*_m_). *Y*-axis values that are greater than one show increased frequencies of *B*_2_ under tight linkage, and values less than one denote reduced frequencies. The dashed curve shows the overall frequency of the deleterious mutation in the population: (*x*_2_ + *x*_4_)/*p*_null_, based on equations (3) and (4b). The black curve shows the frequency of *B*_2_ on haplotypes that carry a male-beneficial allele: (*x*_2_/*q*)/*p*_null_, based on equations (1a) and (4b). The gray curve shows the frequency of *B*_2_ on haplotypes carrying a female-beneficial allele: [*x*_2_/(1 – *q*)]/*p*_null_, based on equations (1b) and (4b). Circles present the results of exact, deterministic simulations under tight linkage, with parameters *u*_f_ = *u*_m_ = 10^−5^, *t*_m_ = 0, and *t*_f_ = 0.1.

The allele frequency difference between adults of each sex is:

$$\delta_{\text{hap}}={\hat{q}}_{\text{m}}-{\hat{q}}_{\text{f}}=\frac{2s_{\text{m}}{\hat{q}}_{\text{hap}}\left(1-{\hat{q}}_{\text{hap}}\right)}{{\bar{w}}_{\text{m}}}=\frac{2s_{\text{f}}{\hat{q}}_{\text{hap}}\left(1-{\hat{q}}_{\text{hap}}\right)}{{\bar{w}}_{\text{f}}},$$ (4b)

where ${\bar{w}}_{\text{m}}=1-\left(1-{\hat{q}}_{\text{hap}}\right)s_{\text{m}}$, ${\bar{w}}_{\text{f}}=1-{\hat{q}}_{\text{hap}}s_{\text{f}}$, and $\hat{q_{j}}$ represents the frequency of *A*_m_ within adults of sex *j* (*j* = {*m*, *f*}). The maximum frequency difference between adults is *δ*_hap_ = 1, which occurs when *s*_m_ = *s*_f_ = 1. The allele frequency difference between adult females and males increases as the equilibrium for each allele approaches equality (${\hat{q}}_{\text{hap}}$→ 0.5, as *s*_m_ → *s*_f_), and as the strength of selection within each sex increases. Increasing *δ*_hap_ leads to increasingly pronounced differences in the mutation load between haplotypes that carry different sexually antagonistic alleles (Figure 1).

#### Sexually antagonistic variation in diploids:

In diploids, the pattern of genetic dominance at a sexually antagonistic locus greatly impacts the parameter space for balanced polymorphism (Kidwell *et al.* 1977; Fry 2010), along with the magnitude of allele frequency differences between adult females and males (as shown below). Following previous models (Fry 2010; Connallon and Clark 2012, 2013), we focus on two idealized versions of dominance among sexually antagonistic alleles: (1) an additive effects model, where the fitness of heterozygotes of each sex is intermediate between homozygote fitnesses (*k*_f_ = *k*_m_ = 1/2 in Table 1); and (2) a strong dominance reversal model, in which the sexually antagonistic allele that is beneficial for each sex has a dominant fitness effect within that sex (*k*_m_ = *k*_f_ = 0 in Table 1; see Barson *et al.* 2015 for an empirical example).

For the additive model, the equilibrium for the male-beneficial allele under balancing selection is identical to that of the haploid model (${\hat{q}}_{\text{add}}={\hat{q}}_{\text{hap}}$). The allele frequency difference between adult females and males is:

$$\delta_{add}=\frac{2{\bar{w}}_{\text{f}}}{s_{\text{f}}}\left(\sqrt{1+\frac{s_{\text{f}}s_{\text{m}}{\hat{q}}_{\text{hap}}\left(1-{\hat{q}}_{\text{hap}}\right)}{{\bar{w}}_{\text{f}}{\bar{w}}_{\text{m}}}-1}\right)=\frac{2{\bar{w}}_{\text{m}}}{s_{\text{m}}}\left(\sqrt{1+\frac{s_{\text{f}}s_{\text{m}}{\hat{q}}_{\text{hap}}\left(1-{\hat{q}}_{\text{hap}}\right)}{{\bar{w}}_{\text{f}}{\bar{w}}_{\text{m}}}-1}\right),$$ (5)

which reduces to $\delta_{\text{add}}=s_{\text{f}}{\hat{q}}_{\text{hap}}\left(1-{\hat{q}}_{\text{hap}}\right)/{\bar{w}}_{\text{f}}=s_{\text{m}}{\hat{q}}_{\text{hap}}\left(1-{\hat{q}}_{\text{hap}}\right)/{\bar{w}}_{\text{m}}$ when sexually antagonistic selection is not too strong (*e.g.*, when *s*_f_, *s*_m_ < 1/2). The maximum allele frequency difference between diploid sexes under the additive model is *δ*_add_ = (√2 – 1) ≈ 0.414.

The parameter space for balancing selection increases under dominance reversal conditions within diploids (*e.g.*, *k*_f_ = *k*_m_ < 1/2; Kidwell *et al.* 1977; Fry 2010). For the complete dominance reversal scenario (*k*_f_ = *k*_m_ = 0), the equilibrium frequency of the male-beneficial allele is approximately:

$${\hat{q}}_{\text{DR}}\approx \frac{s_{\text{m}}}{s_{\text{f}}+s_{\text{m}}},$$ (6a)

and the allele frequency difference between adult males and females is:

$$\delta_{\text{DR}}\approx \frac{2s_{\text{m}}\hat{q}{\left(1-\hat{q}\right)}^{2}}{1-{\left(1-\hat{q}\right)}^{2}s_{\text{m}}}\approx \frac{2s_{\text{f}}{\hat{q}}^{2}\left(1-\hat{q}\right)}{1-{\hat{q}}^{2}s_{\text{f}}}.$$ (6b)

Equation (6b) is accurate unless sexually antagonistic selection is very strong (*i.e.*, the approximation works well for selection coefficients up to roughly *s*_m_, *s*_f_ ∼0.5), and otherwise tends to slightly overestimate allele frequency difference between adult sexes. The maximum for *δ*_DR_ is roughly one third, which falls below the maxima for both the haploid and additive diploid models of antagonistic selection.

#### Polymorphism in haploids *vs.* diploids:

Allele frequency differences between adult sexes at the sexually antagonistic locus are often less pronounced in diploids than haploids. For the additive fitness model [contrasting equations (4b) and (5)], allele frequency differences between adult males and females will always be at least twofold higher in haploids than diploids for each parameter set that maintains a balanced polymorphism. Under weak selection, 2*δ*_add_ ≈ *δ*_hap_, and the discrepancy between *δ*_add_ and *δ*_hap_ further increases with the strength of antagonistic selection. Thus, sex-biased mutation loads that accumulate near sexually antagonistic alleles will tend to be greater in haploids than diploids.

**Figure 2:**
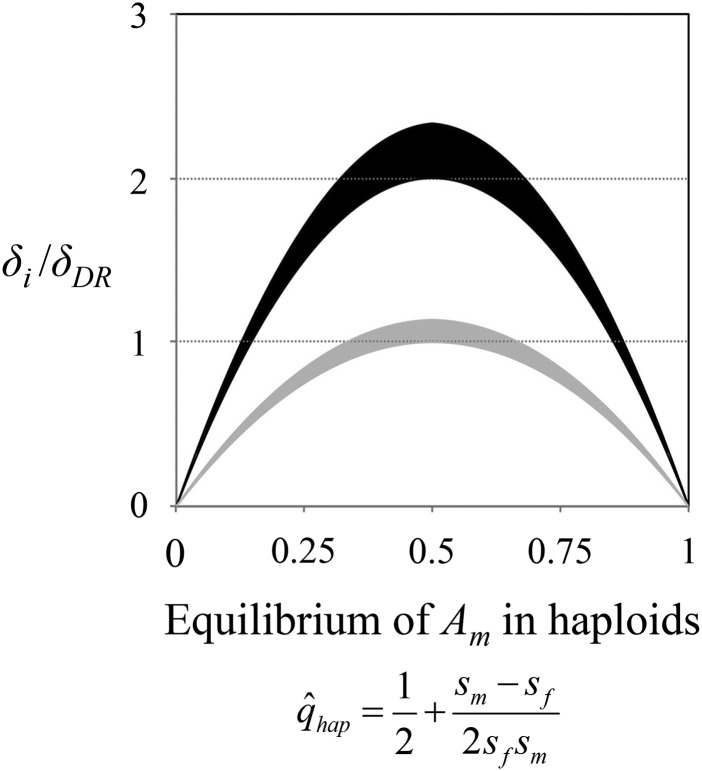
Allele frequency differences between adult females and males at a sexually antagonistic locus. The *x*-axis shows the equilibrium frequency of a male-beneficial allele under the haploid model of selection, which at equilibrium is *q*_hap_ = (*s*_m_ – *s*_f_ + *s*_m_*s*_f_)/(2*s*_m_*s*_f_). The *y*-axis shows the frequency difference of *A*_m_ between adult sexes, scaled to the diploid dominance reversal model [*δ*_DR_ from equation (6b)]. The black shaded region represents values of *δ*_ha_*_p_*/*δ*_DR_, with the upper border of the black shaded area representing strong selection [max(*s*_m_, *s*_f_) → 0.5], and the lower border representing weak selection [max(*s*_m_, *s*_f_) → 0]. The gray shaded region represents values of *δ*_add_/*δ*_DR_, with the upper border of the gray shaded area representing strong selection [max(*s*_m_, *s*_f_) → 0.5], and the lower border representing weak selection [max(*s*_m_, *s*_f_) → 0]. Results are based on equations (4) to (6).

Adult allele frequency differences at the sexually antagonistic locus are also greater in haploids than diploids with dominance reversal (*k*_m_ = *k*_f_ = 0) (Figure 2), unless the selection coefficients are asymmetric between the sexes. As the strength of sexually antagonistic selection becomes increasingly asymmetric, equilibrium heterozygosity at the sexually antagonistic locus declines rapidly in the haploid and additive diploid models (*i.e.*, the equilibria ${\hat{q}}_{\text{hap}}$ and ${\hat{q}}_{\text{dip}}$ approach zero or one as *s*_m_ – *s*_f_ deviates from zero). In contrast, under asymmetric sexually antagonistic selection, substantial heterozygosity is still likely under dominance reversal conditions. When sexually antagonistic selection is weak (*s*_m_, *s*_f_ << 1), *δ*_DR_ > *δ*_hap_ when min(${\hat{q}}_{\text{hap}}$, 1 – ${\hat{q}}_{\text{hap}}$) < ∼0.15, and stronger selection lowers this threshold (Figure 2). A qualitatively similar pattern is observed when contrasting allele frequency differences between the sexes under the additive and dominance reversal scenarios within diploids (Figure 2). In this case, however, *δ*_DR_ > *δ*_add_ is expected under most parameter conditions, with exceptions occurring when selection coefficients at the sexually antagonistic locus are nearly identical between the sexes.

**Figure 3:**
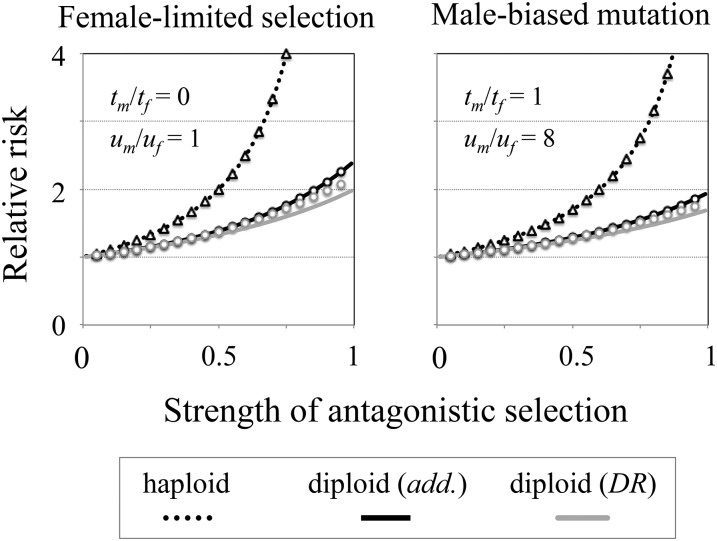
The relative risk of a deleterious mutation on haplotypes that carry a male-beneficial *vs.* a female-beneficial sexually antagonistic allele. Results show the relative enrichment of a deleterious allele on haplotypes that carry the sexually antagonistic allele *A*_m_. The left panel shows the accumulation of a deleterious mutation with female-limited effect (*t*_m_ = 0; *t*_f_ = 0.1) under sexually symmetric mutation (*u*_f_ = *u*_m_ = 10^−5^). The right panel shows the accumulation of a symmetrically deleterious mutation (*t*_m_ = *t*_f_ = 0.1) under male-biased mutation (*u*_f_ = 10^−5^; *u*_m_/*u*_f_ = 8). Sexually antagonistic selection is symmetric between the sexes (*s*_m_ = *s*_f_; *q*_hap_ = *q*_add_ = *q*_DR_ = 0.5). Each curve is based on the general equation (2) (with higher terms of δ included), and the haploid or diploid model expressions in equations (4) to (6). Triangles show the results of deterministic simulations under the haploid model of inheritance; circles show simulation results under diploid inheritance, for each of the dominance scenarios considered.

Sexually antagonistic allele frequency differentiation between adult females and males predicts the degree of mutation accumulation at genes that are linked to sexually antagonistic loci (in terms of the overall frequency of *B*_2_, and the relative enrichment on haplotypes that carry male- or female-beneficial sexually antagonistic alleles). For example, when the strength of sexually antagonistic selection is similar between sexes (*e.g.*, *s*_f_ = *s*_m_), sex-bias in genetic load (*i.e.*, skew in the relative risk of carrying a deleterious allele between haplotypes) is more pronounced in haploids than diploids. Figure 3 illustrates this outcome both due to sex-limited deleterious effects and sex-biased mutation rates, and that these effects can increase strongly with the strength of sexually antagonistic selection under haploidy or diploidy. In contrast, as sexually antagonistic selection becomes increasingly asymmetric between the sexes, balancing selection at a sexually antagonistic locus, as well as mutation accumulation at nearby, linked loci, will be most pronounced under dominance reversal conditions, as apply within diploids.

### Effects of incomplete linkage

**Figure 4:**
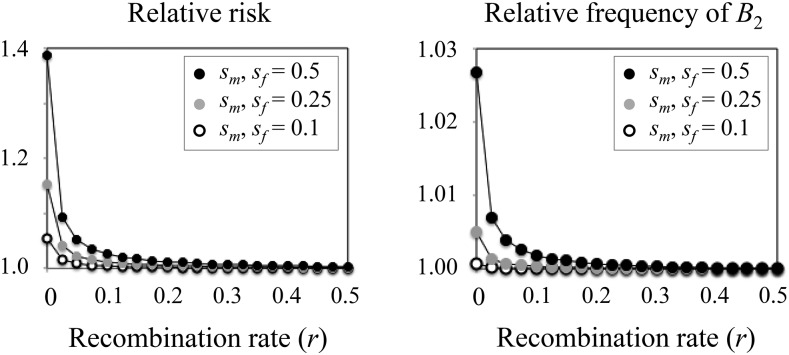
Mutation accumulation under diploid selection and arbitrary linkage between loci. The left panel shows the relative risk of a deleterious mutation on haplotypes that carry a male-beneficial *vs.* a female-beneficial sexually antagonistic allele (as in Figure 3). The right panel displays the equilibrium frequency of a deleterious allele relative to its expected frequency in the absence of sexually antagonistic selection at the *A* locus (as in Figure 1). Results are based on deterministic forward simulations, using recursions from the Appendix. Both panels show the accumulation of a partially recessive (*h* = 0.2) deleterious mutation with female-limited effect (*t*_m_ = 0; *t*_f_ = 0.1), and under sexually symmetric mutation (*u*_f_ = *u*_m_ = 10^−5^). The sexually antagonistic locus evolves under balancing selection with additive and symmetrical fitness effects between the sexes (*s*_m_ = *s*_f_ = 0.1, 0.25 or 0.5; *k*_m_ = *k*_f_ = 1/2 within Table 1).

Figure 4 illustrates that the effects described above diminish rapidly with increasing recombination. Both strong sexually antagonistic selection (*s*_m_ = *s*_f_ = 0.5) and tight linkage to the deleterious locus are required to appreciably increase the frequency of the deleterious allele (the example in the figure exhibits a mild frequency increase of *B*_2_ even under tight linkage; right panel). In contrast, when selection on sexually antagonistic alleles is sufficiently strong, the relative risk of inheriting deleterious alleles can substantially differ between haplotypes that carry male- *vs.* female-benefit alleles, even under incomplete linkage between loci. Nevertheless, relative risk does decay rapidly away from the sexually antagonistic locus, with enrichment of deleterious alleles most likely at small recombinational distances (*e.g.*, *r* = 0.05 or less; left panel).

## Discussion

Opportunities for maintaining sexually antagonistic polymorphisms are theoretically predicted to increase when sexually antagonistic selection is strong, when the strength of antagonistic selection is relatively symmetrical between the sexes, and when patterns of genetic dominance for fitness are sex-specific (as in the dominance reversal condition outlined above; see Kidwell *et al.* 1977; Patten and Haig 2009; Fry 2010; Jordan and Charlesworth 2012; Connallon and Clark 2012, 2014; Arnqvist *et al.* 2014). Recent extensions of this single-locus theory suggest that genetic systems should also evolve to meet conditions that favor the maintenance of sexually antagonistic polymorphisms. For example, sexually antagonistic selection generates patterns of linkage disequilibrium that effectively increase the net strength of selection across tightly linked pairs of sexually antagonistic loci (*e.g.*, Patten *et al.* 2010). Sexual antagonism also favors the evolution of genetic modifiers of sex-specific dominance, which stabilize balanced polymorphisms at sexually antagonistic loci (Spencer and Priest 2016).

Our results demonstrate that the evolutionary conditions that are most conducive for maintaining sexually antagonistic polymorphisms also promote the accumulation of deleterious mutations. Sexually antagonistic variation inflates the overall frequencies of deleterious mutations at genes that are tightly linked to sexually antagonistic loci, typically to greatest effect in haploid species. Moreover, and to a greater degree, haplotypes that carry male-benefit *vs.* female-benefit sexually antagonistic alleles will differ in their relative loads of sex-specific mutations. Male-beneficial haplotypes become enriched for mutations that preferentially harm female fitness, whereas female-benefit haplotypes harbor predominantly male-harming deleterious alleles. This effect amplifies the fitness costs of sexually antagonistic alleles when they are inherited by the “wrong” sex. Females (or males) that inherit male-benefit (female-benefit) alleles will simultaneously inherit an increased linked load of female-harming (male-harming) deleterious mutations. This symmetrical packaging of female- and male-harming alleles on distinct haplotypes may be somewhat offset by male-biased mutation rates, which unequivocally increase the load of mutations carried on haplotypes with male-benefit sexually antagonistic alleles.

Although we framed our models in terms of separate-sexed species, our results should equally apply to obligate outcrossing hermaphrodite species given assumptions regarding the fertility of matings between different genotypes (Bodmer 1965; Jordan and Connallon 2014; Tazzyman and Abbott 2015). In this case, both sexually antagonistic and deleterious alleles impact female and male sex *functions* within individuals (*e.g.*, pollen or ovule production in plants). Self-fertilization in hermaphrodite populations can potentially impact our results in three ways: (1) by decreasing the potential for sexually antagonistic polymorphism (Jordan and Connallon 2014; Tazzyman and Abbott 2015); (2) by facilitating purging of partially or completely recessive deleterious alleles (Charlesworth and Charlesworth 1987); and (3) by decreasing the effective recombination rate between sexually antagonistic and deleterious loci. The net outcome of these contrasting forces for genetic load awaits investigation.

The vast majority of functional loci in the genome evolve under purifying selection (Eyre-Walker and Keightley 2007), and consequently, our predictions should apply to any genomic region that is linked to a polymorphic sexually antagonistic locus. Although strong sexually antagonistic selection is necessary to drastically shift the equilibrium frequency of a single deleterious mutation (*i.e.*, relative to predictions from standard theory of mutation-selection balance; *e.g.*, Haldane 1937; Crow and Kimura 1970), weak sexual antagonism may nevertheless generate modest shifts in the deleterious allele frequencies across many linked sites. Thus, the cumulative mutation burden on sexually antagonistic haplotypes may still be appreciable. Yet strong selection may often be plausible for individual cases of sexually antagonistic polymorphism. Instances of strong sexually antagonistic polymorphism have been implied in several study systems, including delicate skinks (Forsman and Shine 1995), collared flycatchers (Husby *et al.* 2015), Atlantic salmon (Barson *et al.* 2015), and most recently, an inversion polymorphism in the yellow monkey-flower (Lee *et al.* 2016). Such inversions may be particularly prone to the accumulation of deleterious alleles, because recombination can be suppressed between haplotypes comprising many genes (*e.g.*, >1200 genes; Lee *et al.* 2016).

Our results demonstrate that deleterious mutations can, at least in some contexts, accumulate more readily in predominantly haploid than in diploid species. This result runs counter to the traditional view in which purifying selection is enhanced in haploid relative to diploid species, with haploids carrying a reduced genetic load under most conditions of dominance (Crow and Kimura 1965; Otto and Goldstein 1992). It is currently difficult to evaluate the extent of sexually antagonistic genetic variation in haploid species, along with opportunities for the accumulation of deleterious alleles near sexually antagonistic genes. Most empirical work on sexual antagonism has, to date, focused on species with predominantly diploid life cycles, though many dioecious species do have a dominant haploid phase (*e.g.*, Jesson and Garnock-Jones 2012). Such species represent intriguing systems for studying genetic conflicts and their evolutionary genetic consequences (*e.g.*, Immler and Otto 2015). Predictions from the current study should hopefully provide additional motivation for empirical research on sexual antagonism in species with predominantly haploid life cycles.

The enrichment of deleterious alleles near sexually antagonistic loci represents one of several contexts of mutation accumulation that should differentially affect subregions of each sexual genome. Three forms of linked selection are particularly relevant to the evolutionary processes that we have emphasized. First, genes with sex-limited transmission patterns – *i.e.*, those on the W or Y chromosome, and genes encoded by the mitochondrial genome – naturally accumulate mutations with sex-biased fitness effects. Such loci primarily respond to selection in only one of the two sexes, and can fix mutations that harm the other (Frank and Hurst 1996; Charlesworth *et al.* 2014).

Second, obligate outcrossing plant species typically exhibit high levels of genetic diversity at self-incompatibility (SI) loci. These SI loci promote heterozygosity at sites to which they are physically linked, and thereby shelter recessive deleterious mutations from purifying selection (see Glémin *et al.* 2001; Stift *et al.* 2013; also see Strobeck 1972 for consideration in heterostylous plants, and van Oosterhout 2009, for comparable models at MHC loci). The ultimate consequence of balancing selection at SI loci is the accumulation of mutations at nearby genes – a similar outcome to the one outlined here, though the underlying mechanisms of selection and genetic transmission differ.

Finally, tight physical linkage can itself promote the accumulation of deleterious alleles by reducing the overall efficacy of natural selection relative to genetic drift (*e.g.*, due to Hill–Robertson interference between linked loci under selection; Hill and Robertson 1966; Charlesworth 2012). Genomic variation in recombination rates gives rise to heterogeneity across the genome for the efficacy of natural selection, and genomic regions with little or no recombination are particularly prone to functional decay – through the accumulation of deleterious mutations and a failure to retain beneficial ones. Linkage disequilibrium, arising from selection among linked loci, can also promote the evolution of increased or decreased recombination (Otto and Lenormand 2002), including sexually dimorphic recombination rates (Lenormand 2003; Lenormand and Dutheil 2006). The form of linkage disequilibrium predicted here – of sexually antagonistic alleles cosegregating with mutations that carry sex-biased fitness costs – provides an intriguing context for the evolution of sexually dimorphic recombination rates (as observed in many animal and plant taxa; Lenormand and Dutheil 2006), for which sex differences in selection and haploidy have both been shown to play important roles (Lenormand 2003).

## Appendix

### General model for haploid selection

Under haploid selection, we follow the frequency of four haplotypes. We start with the gametes (sperm and eggs) among the surviving adults of an arbitrary generation. Let the haplotype frequencies in eggs and sperm be:

$$x_{1e}=\left[A_{m}B_{1}\right];x_{2e}=\left[A_{m}B_{2}\right];x_{3e}=\left[A_{f}B_{1}\right];x_{4e}=\left[A_{f}B_{2}\right]$$

$$x_{1s}=\left[A_{m}B_{1}\right];x_{2s}=\left[A_{m}B_{2}\right];x_{3s}=\left[A_{f}B_{1}\right];x_{4s}=\left[A_{f}B_{2}\right]$$

where *x_i_*_e_ refers to the frequency of the *i*th haplotype in eggs and *x_i_*_s_ represents the frequency of the *i*th haplotype in sperm. Sperm and eggs combine at random to produce diploid zygotes, and then immediately undergo meiosis to produce haploid offspring. The frequency of each haplotype in offspring (which will be the same in male and female offspring) is:

$$\begin{array}{l} f_{1}=\frac{1}{2}\left(x_{\text{1}e}+x_{1s}\right)-\frac{r}{2}\left(x_{1e}x_{4s}+x_{4e}x_{1s}-x_{2e}x_{\text{3}s}-x_{\text{3}e}x_{\text{2}s}\right)\\ f_{2}=\frac{1}{2}\left(x_{\text{2}e}+x_{\text{2}s}\right)+\frac{r}{2}\left(x_{\text{1}e}x_{\text{4}s}+x_{4e}x_{\text{1}s}-x_{\text{2}e}x_{\text{3}s}-x_{\text{3}e}x_{\text{2}s}\right)\\ f_{3}=\frac{1}{2}\left(x_{\text{3}e}+x_{\text{3}s}\right)+\frac{r}{2}\left(x_{\text{1}e}x_{\text{4}s}+x_{\text{4}e}x_{\text{1}s}-x_{\text{2}e}x_{\text{3}s}-x_{\text{3}e}x_{\text{2}s}\right)\\ f_{4}=\frac{1}{2}\left(x_{\text{4}e}+x_{\text{4}s}\right)-\frac{r}{2}\left(x_{\text{1}e}x_{4s}+x_{4e}x_{\text{1}s}-x_{\text{2}e}x_{\text{3}s}-x_{3e}x_{\text{2}s}\right) \end{array}$$

where *r* represents the crossover rate between the *A* and *B* locus (0 < *r* < 1/2).

After sex-specific viability selection, the haplotype frequencies in adult females are:

$$\begin{array}{l} x_{\text{1}f}=\frac{f_{1}\left(1-s_{f}\right)}{{\bar{w}}_{f}}\\ x_{2f}=\frac{f_{2}\left(1-s_{f}\right)\left(1-t_{f}\right)}{{\bar{w}}_{f}}\\ x_{3f}=\frac{f_{3}}{{\bar{w}}_{f}}\\ x_{4f}=\frac{f_{4}\left(1-t_{f}\right)}{{\bar{w}}_{f}} \end{array}$$

The haplotype frequencies in males are:

$$\begin{array}{l} x_{1m}=\frac{f_{1}}{{\bar{w}}_{m}};\;\\ x_{2m}=\frac{f_{2}\left(1-t_{m}\right)}{{\bar{w}}_{m}};\;\\ x_{3m}=\frac{f_{3}\left(1-s_{m}\right)}{{\bar{w}}_{m}};\;\\ x_{4m}=\frac{f_{4}\left(1-s_{m}\right)\left(1-t_{m}\right)}{{\bar{w}}_{m}} \end{array}$$

The mean relative fitnesses of females and males are:

$${\bar{w}}_{f}=f_{1}\left(1-s_{f}\right)+f_{2}\left(1-s_{f}\right)\left(1-t_{f}\right)+f_{3}+f_{4}\left(1-t_{f}\right)$$

and

$${\bar{w}}_{m}=f_{1}+f_{2}\left(1-t_{m}\right)+f_{3}\left(1-s_{m}\right)+f_{4}\left(1-s_{m}\right)\left(1-t_{m}\right)$$

The life cycle is completed after accounting for mutation in the eggs and sperm that are produced by surviving adults in the population. The mutation rate to the deleterious allele (with no back-mutation) is *u*_m_ in males and *u*_f_ in females, per gamete. Thus, the full recursion is:

$${x^{\prime }}_{1e}^{}=x_{1f}\left(1-u_{f}\right)$$

$${x^{\prime }}_{2e}^{}=x_{1f}u_{f}+x_{2f}$$

$${x^{\prime }}_{3e}^{}=x_{3f}\left(1-u_{f}\right)$$

$${x^{\prime }}_{4e}^{}=x_{3f}u_{f}+x_{4f}$$

$${x^{\prime }}_{1s}^{}=x_{1m}\left(1-u_{m}\right)$$

$${x^{\prime }}_{2s}^{}=x_{1m}u_{m}+x_{2m}$$

$${x^{\prime }}_{3s}^{}=x_{3m}\left(1-u_{m}\right)$$

$${x^{\prime }}_{4s}^{}=x_{3m}u_{m}+x_{4m}$$

### General model for diploid selection

The diploid model follows the same basic form as the haploid model, except that organisms spend the life cycle in diploid state. As before, eggs and sperm of adults combine at random to form diploid offspring. This is followed by viability selection, then gametogenesis in surviving adults, and mutations in their gametes.

Viability selection occurs in a sex-specific manner to produce the following haplotype frequencies in adults (modified from Connallon and Clark 2010). Haplotype frequencies in adult females are:

$$\begin{array}{c} x_{1f}=\frac{2x_{1e}x_{1s}f_{11}+\left(x_{1e}x_{2s}+x_{2e}x_{1s}\right)f_{12}+\left(x_{1e}x_{3s}+x_{3e}x_{1s}\right)f_{21}+\left(x_{1e}x_{4s}+x_{4e}x_{1s}\right)f_{22}}{2{\bar{w}}_{f}}\\ -\frac{r\left(x_{1e}x_{4s}+x_{4e}x_{1s}-x_{2e}x_{3s}-x_{3e}x_{2s}\right)f_{22}}{2{\bar{w}}_{f}}\\ x_{2f}=\frac{2x_{2e}x_{2s}f_{13}+\left(x_{1e}x_{2s}+x_{2e}x_{1s}\right)f_{12}+\left(x_{2e}x_{4s}+x_{4e}x_{2s}\right)f_{23}+\left(x_{2e}x_{3s}+x_{3e}x_{2s}\right)f_{22}}{2{\bar{w}}_{f}}\\ +\frac{r\left(x_{1e}x_{4s}+x_{4e}x_{1s}-x_{2e}x_{3s}-x_{3e}x_{2s}\right)f_{22}}{2{\bar{w}}_{f}}\\ x_{3f}=\frac{2x_{3e}x_{3s}f_{31}+\left(x_{1e}x_{3s}+x_{3e}x_{1s}\right)f_{21}+\left(x_{3e}x_{4s}+x_{4e}x_{3s}\right)f_{32}+\left(x_{2e}x_{3s}+x_{3e}x_{2s}\right)f_{22}}{2{\bar{w}}_{f}}\\ +\frac{r\left(x_{1e}x_{4s}+x_{4e}x_{1s}-x_{2e}x_{3s}-x_{3e}x_{2s}\right)f_{22}}{2{\bar{w}}_{f}}\\ x_{4f}=\frac{2x_{4e}x_{4s}f_{33}+\left(x_{2e}x_{4s}+x_{4e}x_{2s}\right)f_{23}+\left(x_{3e}x_{4s}+x_{4e}x_{3s}\right)f_{32}+\left(x_{1e}x_{4s}+x_{4e}x_{1s}\right)f_{22}}{2{\bar{w}}_{f}}\\ -\frac{r\left(x_{1e}x_{4s}+x_{4e}x_{1s}-x_{2e}x_{3s}-x_{3e}x_{2s}\right)f_{22}}{2{\bar{w}}_{f}} \end{array}$$

where $2{\bar{w}}_{f}$ is the sum of the numerators in *x*_1_*_f_*, *x*_1_*_f_*, *x*_3_*_f_*, and *x*_4_*_f_*, and values of *f_ij_* are given in Table A1. The haplotype frequencies in adult males are:

$$\begin{array}{c} x_{1m}=\frac{2x_{1e}x_{1s}m_{11}+\left(x_{1e}x_{2s}+x_{2e}x_{1s}\right)m_{12}+\left(x_{1e}x_{3s}+x_{3e}x_{1s}\right)m_{21}+\left(x_{1e}x_{4s}+x_{4e}x_{1s}\right)m_{22}}{2{\bar{w}}_{m}}\\ -\frac{r\left(x_{1e}x_{4s}+x_{4e}x_{1s}-x_{2e}x_{3s}-x_{3e}x_{2s}\right)m_{22}}{2{\bar{w}}_{m}}\\ x_{2m}=\frac{2x_{2e}x_{2s}m_{13}+\left(x_{1e}x_{2s}+x_{2e}x_{1s}\right)m_{12}+\left(x_{2e}x_{4s}+x_{4e}x_{2s}\right)m_{23}+\left(x_{2e}x_{3s}+x_{3e}x_{2s}\right)m_{22}}{2{\bar{w}}_{m}}\\ +\frac{r\left(x_{1e}x_{4s}+x_{4e}x_{1s}-x_{2e}x_{3s}-x_{3e}x_{2s}\right)m_{22}}{2{\bar{w}}_{m}}\\ x_{3m}=\frac{2x_{3e}x_{3s}m_{31}+\left(x_{1e}x_{3s}+x_{3e}x_{1s}\right)m_{21}+\left(x_{3e}x_{4s}+x_{4e}x_{3s}\right)m_{32}+\left(x_{2e}x_{3s}+x_{3e}x_{2s}\right)m_{22}}{2{\bar{w}}_{m}}\\ +\frac{r\left(x_{1e}x_{4s}+x_{4e}x_{1s}-x_{2e}x_{3s}-x_{3e}x_{2s}\right)m_{22}}{2{\bar{w}}_{m}}\\ x_{4m}=\frac{2x_{4e}x_{4s}m_{33}+\left(x_{2e}x_{4s}+x_{4e}x_{2s}\right)m_{23}+\left(x_{3e}x_{4s}+x_{4e}x_{3s}\right)m_{32}+\left(x_{1e}x_{4s}+x_{4e}x_{1s}\right)m_{22}}{2{\bar{w}}_{m}}\\ -\frac{r\left(x_{1e}x_{4s}+x_{4e}x_{1s}-x_{2e}x_{3s}-x_{3e}x_{2s}\right)m_{22}}{2{\bar{w}}_{m}} \end{array}$$

where $2{\bar{w}}_{m}$ is the sum of the numerators in *x*_1_*_m_*, *x*_1_*_m_*, *x*_3_*_m_*, and *x*_4_*_m_*, and values of *m_ij_* are given in Table A1.

**Table A1:** Sex-specific fitness per diploid genotype

	*A_m_A_m_*	*A_m_A_f_*	*A_f_A_f_*
*B*_1_*B*_1_	*f*_11_ = 1 – *s_f_*	*f*_21_ = 1 – *k_f_s_f_*	*f*_31_ = 1
	*m*_11_ = 1	*m*_21_ = 1 – *k_m_s_m_*	*m*_31_ = 1 – *s_m_*
*B*_1_*B*_2_	*f*_12_ = (1 – *s_f_*)(1 – *ht_f_*)	*f*_22_ = (1 – *k_f_s_f_*)(1 – *ht_f_*)	*f*_32_ = 1 – *ht_f_*
	*m*_12_ = 1 – *ht_m_*	*m*_22_ = (1 – *k_m_s_m_*)(1 – *ht_m_*)	*m*_32_ = (1 – *s_m_*)(1 – *ht_m_*)
*B*_2_*B*_2_	*f*_13_ = (1 – *s_f_*)(1 – *t_f_*)	*f*_23_ = (1 – *k_f_s_f_*)(1 – *t_f_*)	*f*_33_ = 1 – *t_f_*
	*m*_13_ = 1 – *t_m_*	*m*_23_ = (1 – *k_m_s_m_*)(1 – *t_m_*)	*m*_33_ = (1 – *s_m_*)(1 – *t_m_*)

The life cycle is completed after accounting for mutation in eggs and sperm, leading to:

$${x^{\prime }}_{1e}^{}=x_{1f}\left(1-u_{f}\right)$$

$${x^{\prime }}_{2e}^{}=x_{1f}u_{f}+x_{2f}$$

$${x^{\prime }}_{3e}^{}=x_{3f}\left(1-u_{f}\right)$$

$${x^{\prime }}_{4e}^{}=x_{3f}u_{f}+x_{4f}$$

$${x^{\prime }}_{1s}^{}=x_{1m}\left(1-u_{m}\right)$$

$${x^{\prime }}_{2s}^{}=x_{1m}u_{m}+x_{2m}$$

$${x^{\prime }}_{3s}^{}=x_{3m}\left(1-u_{m}\right)$$

$${x^{\prime }}_{4s}^{}=x_{3m}u_{m}+x_{4m}$$

### Mutation-selection balance approximations for tight linkage

We assume that the mutation rate at the *B* locus is weak relative to purifying selection (*u_m_* + *u_f_* << *t_m_* + *t_f_*) and relative to the strength of selection at the sexually antagonistic locus. Thus, the frequency of the deleterious allele will be maintained at a low frequency in the population, and evolution at the sexually antagonistic locus will be approximately independent of selection at the *B* locus.

The equilibrium frequency of the *A_m_* allele in adult males and females (respectively) is given by ${\hat{q}}_{m}$ and ${\hat{q}}_{f}$. The overall frequency in the population (in offspring) is simply the average of sex-specific adult frequencies: $\hat{q}=\left({\hat{q}}_{m}+{\hat{q}}_{f}\right)/2$, which meets the constraint ${\hat{q}}_{m}>\hat{q}>{\hat{q}}_{f}$. The adult allele frequencies can be used to calculate the relative proportions of male-beneficial alleles that are inherited from fathers and mothers. At equilibrium, these are:

$$\mathrm{Pr}\left(A_{m}|\text{fathers}\right)=\frac{{\hat{q}}_{m}}{2\hat{q}}$$

and

$$\mathrm{Pr}\left(A_{m}|\text{mothers}\right)=\frac{{\hat{q}}_{f}}{2\hat{q}}$$

Likewise, the relative proportions of female-beneficial alleles that were inherited from mothers and fathers are:

$$\mathrm{Pr}\left(A_{f}|\text{mothers}\right)=\frac{1-{\hat{q}}_{f}}{2\left(1-\hat{q}\right)}$$

and

$$\mathrm{Pr}\left(A_{f}|\text{fathers}\right)=\frac{1-{\hat{q}}_{m}}{2\left(1-\hat{q}\right)}$$

When the *B* locus is completely linked to the sexually antagonistic locus, then a fraction of *B* genes in the population completely cosegregates with the male-beneficial allele. These genes spend a larger fraction of time evolving in male bodies than female bodies. The remaining fraction of *B* genes cosegregates with the female-beneficial allele, and carried in female bodies more than male bodies.

We can use the equilibrium frequencies of the sexually antagonistic allele to calculate the effective strength of selection against deleterious mutations on male-beneficial and female-beneficial haplotypes. The strength of purifying selection on male-beneficial haplotypes is proportional to:

$$t_{eff}|A_{m}\propto t_{f}\frac{{\hat{q}}_{f}}{2\hat{q}}+t_{m}\frac{{\hat{q}}_{m}}{2\hat{q}}=\frac{\left(t_{f}{\hat{q}}_{f}+t_{m}{\hat{q}}_{m}\right)}{2\hat{q}}$$

and the strength of selection on female-beneficial haplotypes is proportional to:

$$t_{eff}|A_{f}\propto \frac{t_{f}\left(1-{\hat{q}}_{f}\right)+t_{m}\left(1-{\hat{q}}_{m}\right)}{2\left(1-\hat{q}\right)}$$

Likewise, the effective mutation rates on male- and female-beneficial haplotypes will be:

$$u_{eff}|A_{m}=\frac{\left(u_{f}{\hat{q}}_{f}+u_{m}{\hat{q}}_{m}\right)}{2\hat{q}}$$

and

$$u_{eff}|A_{f}=\frac{u_{f}\left(1-{\hat{q}}_{f}\right)+u_{m}\left(1-{\hat{q}}_{m}\right)}{2\left(1-\hat{q}\right)}$$

Because *u_eff_* | *A_j_* << *t_eff_* | *A_j_*, the equilibrium frequency of the deleterious allele on each haplotype can be approximated as:

$${\hat{f}}_{B}|A_{m}=C\frac{u_{eff}|A_{m}}{t_{eff}|A_{m}}$$

and

$${\hat{f}}_{B}|A_{f}=C\frac{u_{eff}|A_{f}}{t_{eff}|A_{f}}$$

where *C* is a constant that accounts for ploidy level (for selection in haploids, *C* = 1; in diploids *C* = 1/*h*). Therefore, the frequencies of the four haplotypes will be:

$$\begin{array}{l} {\hat{x}}_{1}=\hat{q}\left(1-{\hat{f}}_{B}|A_{m}\right)\\ {\hat{x}}_{2}=\hat{q}\left({\hat{f}}_{B}|A_{m}\right)\\ {\hat{x}}_{3}=\left(1-\hat{q}\right)\left(1-{\hat{f}}_{B}|A_{f}\right)\\ {\hat{x}}_{4}=\left(1-\hat{q}\right)\left({\hat{f}}_{B}|A_{f}\right) \end{array}$$

These expressions lead to equations (1) to (3) in the main text.
